# Functional roles of an engineer species for coastal benthic invertebrates and demersal fish

**DOI:** 10.1002/ece3.2857

**Published:** 2017-06-15

**Authors:** Aurélie Chaalali, Anik Brind'Amour, Stanislas F. Dubois, Hervé Le Bris

**Affiliations:** ^1^ Ecology and Models Applied to Fishery Resources IFREMER Nantes France; ^2^ ESE, Ecology and Ecosystem Health Agrocampus Ouest INRA Rennes France; ^3^ LEBCO Brittany Center IFREMER Plouzané France

**Keywords:** coastal nurseries, fish community, *Haploops nirae*, isotopic diversity indices, stable isotopes

## Abstract

Through their tissues or activities, engineer species create, modify, or maintain habitats and alter the distribution and abundance of many plants and animals. This study investigates key ecological functions performed by an engineer species that colonizes coastal ecosystems. The gregarious tubiculous amphipod *Haploops nirae* is used as a biological model. According to previous studies, the habitat engineered by *H. nirae* (i.e., *Haploops* habitat) could provide food and natural shelter for several benthic species such as benthic diatoms belonging to the gender *Navicula*, the micrograzer *Geitodoris planata,* or the bivalve *Polititapes virgineus*. Using data from scientific surveys conducted in two bays, this study explored whether (1) the *Haploops* sandy‐mud community modifies invertebrate and ichthyologic community structure (diversity and biomass); (2) *H. nirae* creates a preferential feeding ground; and (3) this habitat serves as a refuge for juvenile fish. Available Benthic Energy Coefficients, coupled with more traditional diversity indices, indicated higher energy available in *Haploops* habitat than in two nearby habitats (i.e., *Sternaspis scutata* and *Amphiura filiformis/Owenia fusiformis* habitats). The use of isotopic functional indices (IFIs) indicated (1) a higher functional richness in the *Haploops* habitat, related to greater diversity in food sources and longer food chains; and (2) a higher functional divergence, associated with greater consumption of a secondary food source. At the invertebrate‐prey level, IFIs indicated little specialization and little trophic redundancy in the engineered habitat, as expected for homogenous habitats. Our results partly support empirical knowledge about engineered versus nonengineered habitats and also add new perspectives on habitat use by fish and invertebrate species. Our analyses validated the refuge‐area hypothesis for a few fish species. Although unique benthic prey assemblages are associated with *Haploops* habitat, the hypothesis that it is a preferential feeding area was not verified. However, specialist feeding behavior was observed for predators, which calls for further investigation.

## 1 Introduction

Coastal and estuarine systems occupy only approximately 6% of marine surface area but are among the most productive areas on earth (Costanza et al., 1997). These systems are associated with strong economic and social issues as they have high human population densities while providing numerous ecosystem functions and services (MEA, 2005). Regarding the latter, they are often described as essential nurseries (Pasquaud et al., 2012; Selleslagh & Amara, 2015) because they serve as growth or refuge areas for many species, especially fish and birds (McLuscky & Elliott, 2004; Pasquaud et al., 2012; Riley, Symonds, & Woolner, 1981; Rochard, Castelnaud, & Lepage, 1990). Degradation and destruction of these coastal areas may lead to the decline of certain marine species and consequently affect the size of commercial fish stocks (Gibson, Yin, & Robb, 1995; Grousset, Jouanneau, Castaing, Lavaux, & Latouche, 1999; Rochette et al., 2010).

In coastal ecosystems, engineer species often perform key functions (Hooper et al., 2005). Besides corals, many other species are crucial for the functioning of shallow coastal waters, such as kelp (Steneck et al., 2002), *Zostera marina* (Bruno & Bertness, 2001), and tubiculous annelids (Mermillod‐Blondin & Lemoine, 2010). Since the concept of “ecosystem engineer” was first defined in 1994 (Jones, Lawton, & Shachak, 1994; Lawton, 1994), a growing body of literature has focused on engineered habitats and their roles in ecosystems. Jones et al. (1994) distinguished two classes of engineers: autogenic engineers (changing the environment *via* their own tissues) and allogenic engineers (changing the environment by transforming resources from one physical state to another). By creating habitat patches, increasing spatial heterogeneity, and in turn, habitat complexity, they also may modulate species diversity (Crooks, 2002; Hendy, Michie, & Taylor, 2014; Jones, Lawton, & Shachak, 1997; Wright & Jones, 2006). Two responses are usually observed: (1) an increase in species richness, for example, an addition of species strongly associated with the engineer species (Castilla, Lagos, & Cerda, 2004; Wright, Jones, & Flecker, 2002); and (2) changes in species abundances, modifying the evenness of species assemblages (Crooks & Khim, 1999; Thomas, Renaurd, de Meeûs, & Poulin, 1998). According to Hastings et al. (2007), the processes underlying the concept of engineering (1) are multiple; (2) should be discussed from a temporal and spatial perspective; and (3) act at multiple biological levels, that is, from individual to ecosystem levels. For example, at the population level, engineer species can affect both the extinction and (re)colonization of habitat patches, thus influencing genetic diversity (McCauley 1993 in Hastings et al., 2007). At the community level, modified species distribution among habitat patches is observed, with marked consequences for species interactions, trophic niche differentiation, trophic levels (Crain & Bertness, 2006; Erwin, 2005), and food‐web functioning (Sanders et al., 2014).

Shallow marine waters surrounding Brittany (West coast of France) are largely dominated by sandy and muddy soft‐bottom habitats. In several bays and archipelagos, muddy sediments are extensively colonized by a gregarious tubiculous amphipod, *Haploops nirae* (Amphipoda)—often confused with *H. tubicola* (Rigolet, Dubois, Droual, Caisey, & Thiébaut, 2012). This engineer species occurs in high densities and actively modifies the sediment‐water interface through tube‐building and filtering activities (Rigolet, Le Souchu, Caisey, & Dubois, 2011). As a result, it strongly affects the composition and turnover of the associated micro‐ and macro‐fauna and creates refuges for endemic species (Myers, Rigolet, Thiebaut, & Dubois, 2012) and a unique assemblage of species found exclusively in this habitat (Rigolet, Dubois, & Thiébaut, 2013). The two main habitats engineered by *H. nirae* (termed hereafter “*Haploops* habitats”) in the northeastern Atlantic are located on the Brittany coast, in the Bay of Concarneau and the Bay of Vilaine (Ehrhold, Hamon, & Guillaumont, 2006; Glémarec, Le Bris, & Le Guellec, 1986; Rigolet, 2013). Opportunistic video surveys revealed continuous expansion of the two *Haploops* habitats over the last two decades (Baltzer et al., 2014; Ehrhold et al., 2006). To explain the ecological succession of these habitats, it was suggested that *Haploops* colonization of surrounding habitats is facilitated by the presence of the tubiculous polychaete *Maldane glebifex* (Glémarec et al., 1986), which is associated with the *Amphiura filiformis* sandy‐mud habitat. Similar colonization of tubiculous amphipod species has been reported in shallow waters, with profound effects on habitat functioning. A drastic change in bentho‐pelagic couplings and an improvement in benthic habitat quality were reported in Boston Harbor (USA) after colonization by tubiculous amphipod *Ampelisca* spp. (Diaz, Rhoads, Blake, Kropp, & Keay, 1998). This improvement was linked to the species processing large amounts of particulate organic matter, reworking the sediment, and increasing interstitial space oxygenation. Similarly, Mackenzie, Pikanowski, and McMillan (2006) suggested that dense tubiculous amphipod colonies could stabilize sediments, thereby minimizing silt transport, and facilitate colonization and development of suspension‐feeding species. According to Sanders et al. (2014), although engineering pathways can influence one or more species up through the entire food web, engineers that create entirely new habitats have larger impacts on a food web. For example, coral‐reef engineers create reefs that increase the richness of species that depend on shelter from wave action, are corallivores or feed on corallivores (Tribollet & Golubic, 2011). Potential consequences such as trophic niche diversification and trophic feedbacks to the engineers via their predators or competitors may also occur (Jones et al., 2010).

According to Tupper and Boutilier (1995), highly structured habitats may provide refuge for both predators and prey, and consequently strongly influence the survival of juveniles of commercial fish species. Indeed, physically complex structures are common features of engineered benthic habitats. In their review, Hastings et al. (2007) documented different examples showing how engineering activities may protect species found in engineered habitats from abiotic forcing and predation. Hendy et al. (2014), studying dead wood in the intertidal zone of mature mangrove forests tunneled by teredinid bivalves, demonstrated that the tunnels constituted a key low‐tide refuge for many cryptic species (e.g., invertebrates and juvenile fish) which would otherwise be predated by fish or birds.

In this study, we assess the ecological role of the engineer species, *H. nirae*—used here as a biological model—for coastal fish and invertebrate species. We tested (1) whether it modifies the diversity, species composition, and biomass of invertebrates and bentho‐demersal fish species, and whether its engineered habitat creates; (2) a preferential feeding ground (notably with high‐energy prey); and (3) a refuge for juvenile fish. Finally, we discuss the functional roles of *H. nirae*, our model engineer species, in comparison with examples from the literature.

## 2 Materials and Methods

### 2.1 Study area and sampling protocol

**Figure 1:**
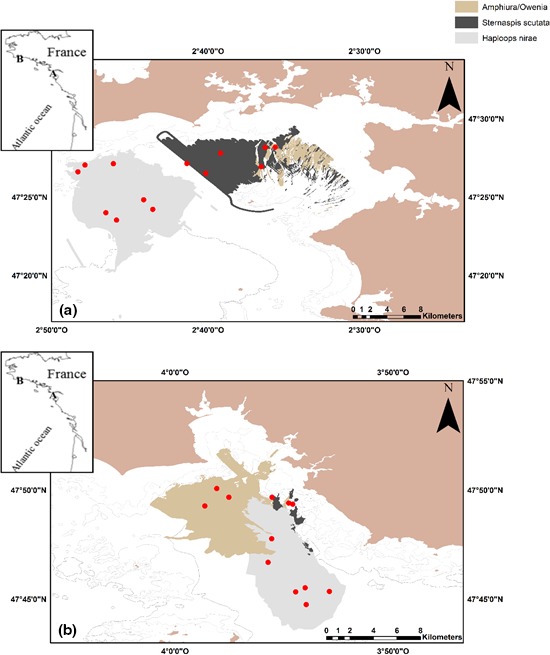
Maps of the two studied bays with local bathymetry with an additional map of France showing their respective locations. (a) Bay of Vilaine. (b) Bay of Concarneau. The three study habitats, that is, *Haploops* habitat, *Amphiura/Owenia* habitat, and *Sternaspis* habitat, are distinguished using a gradient of gray colors. The mean locations of hauls are presented in red circles numbered from 1 to 25

On the Atlantic coast, the Bay of Concarneau and the Bay of Vilaine are recognized as essential nurseries for many exploited fish species, particularly marine bentho‐demersal species (Beck et al., 2001; Le Pape et al., 2003, 2013; Trimoreau, Archambault, Brind'Amour, Guitton, & Le Pape, 2013). This study focuses on these two coastal bays in the northern Bay of Biscay (southern Brittany; Figure 1a,b). The Bay of Vilaine is an open shallow (5–35 m deep) muddy estuarine system covering an area of 230 km^2^ under the influence of the Vilaine River. The Bay of Concarneau, more enclosed by surrounding rocky islets, is 15–35 m deep and has soft muddy bottoms (Ehrhold et al., 2006). These bays have dense habitats of *H. nirae*, which are surrounded by similar soft‐bottom habitats: *Sternaspis scutata* muddy habitat and *A. filiformis*/*Owenia fusiformis* muddy‐sand habitat (Glémarec, 1969; Glémarec et al., 1986; Ehrhold et al., 2006; Rigolet, 2013; Figure 1). These benthic habitats are hereafter referred to as *Haploops*,* Sternaspis,* and *Amphiura/Owenia* habitats, respectively.

Using a preliminary map of the seabed in the two bays, we identified the three aforementioned habitats and selected 12 stations per bay to compare the ecological roles of the *Haploops* habitat (six stations) with the adjacent uncolonized *Sternaspis* and *Amphiura/Owenia* habitats (three stations in each uncolonized habitats). An additional 7th haul was performed in *Haploops* habitat, in the Bay of Vilaine only. Invertebrates and fish species were sampled in August 2009 in each of the two bays using that stratified sampling design. The same design was applied in each bay even though the spatial organization of the three habitats in the two bays largely differed. The sampling locations were arbitrary defined among sampling locations which were deemed trawlable in each bay. A 3‐m beam trawl with a 0.5‐m vertical opening with a 20‐mm stretched mesh in the cod end was used as the sampling device. It was adapted to *Haploops* tubes, having a larger mesh size on the side of the belly (Désaunay, Laffargue & Lobry pers. comm.). Each haul lasted 15 min and covered an area of 4,500–5,000 m^2^.

All fish and benthic invertebrate species were identified to the lowest taxonomic level (usually species), numbered, and weighed to analyze diversity. See Appendix Tables S1 and S2 for a complete list of species. All sampled benthic invertebrate and fish individuals were kept for isotopic analyses (δ^13^C and δ^15^N isotopic composition). Isotopic composition of organic matter sources (i.e., sediment organic matter and particulate organic matter) was also collected for each habitat near the center of each haul.

### 2.2 Data analysis

#### 2.2.1 Fish data selection

To study the role of *H. nirae* in the two coastal bays, we selected fish juveniles only. That selection was performed by choosing a maximal body length corresponding to the age of 2 years for each species (Appendix Table S2). The size limit for each fish species was performed by size‐spectra analyses using a national database from the French Institute for Exploitation of the Sea (IFREMER) or using thresholds from the literature or expert judgments (e.g., Léauté & Mahé, 2008; Ravard, Brind'Amour, & Trenkel, 2014).

#### 2.2.2 Predators/prey definition

In this study, we considered as predators all the fish juveniles and benthic mega‐invertebrates (>1 cm) competing with fish juveniles, whereas the prey were defined according to stomach gut contents analyzed in Tableau, Le Bris, and Brind'Amour (2015). The list compiled by these authors was completed with three species: *Atelecyclus rotundatus*,* Liocarcinus depurator*, and *Ophiocomina nigra*. It is worth noting that depending on individual size, some prey in a habitat may have been considered as predator in another habitat (see Appendix Table S1 for more details).

#### 2.2.3 Calculation of Available Benthic Energy Coefficients

The Available Benthic Energy Coefficient (ABEC) estimates the energetic value of benthic invertebrate prey available to a predator. Thus, ABEC values were only calculated for species considered as prey in this study (Appendix Table S1). ABEC (in kJ·g^−1^·year^−1^) of a prey species *i* is calculated as the product of the prey's mass energy (*E*, in kJ/g), productivity (π, in /year), coefficient of regeneration (*R*, unitless), and coefficient of accessibility (*A*, unitless): ABEC_*i*_ = *E*_*i*_ × π_*i*_ × *R*_*i*_ × *A*_*i*_.

We calculated ABEC values, using coefficients of regeneration and accessibility from Tableau et al. (2015) and wet mass and mass energies from our study. For the three additional species, we obtained mass energies from conversion factors of Brey, Mueller‐Wiegmann, Zittier, and Hagen (2010) and productivities from Brey (2012). We also assumed that both bays had a mean temperature of 13.9°C. The coefficients of regeneration and accessibility were then multiplied by the respective biomass of each prey taxon to obtain an ABEC per unit area for a given habitat (expressed in kJ·year^−1^·km^−2^). Finally, once multiplied by total habitat area, ABECs were expressed at the habitat scale (in kJ/year; Table 1).

**Table 1:** Details of the isotopic functional indices (IFIs) used in this study with their respective codes, full names, and ecological definitions

IFI code	Name	Ecological description	Food web	Trophic niche
SEA	Standard ellipse area	Functional richness; Calculated from the variance and covariance of the δ^13^C and δ^15^N data (Jackson, Inger, Parnell, & Bearhop, 2011)	X	X
NR	δ^15^N range	Proxy of chain length; Calculated as the maximal difference in δ^15^N signatures	X	X
IDiv	Isotopic divergence	Trophic divergence; Species deviance from the mean distance to the center of gravity weighted by relative biomass (Rigolet et al., 2015)	X	X
IDis	Isotopic dispersion	Isotopic signatures dispersion; Weighted mean distance of species to the community weighted center of gravity (Rigolet et al., 2015)		X
IEve	Isotopic eveness	Patchiness/regularity in biomass distribution; Evenness of biomasses distribution in the minimum spanning tree (Rigolet et al., 2015)		X

IFIs have been calculated using species and sources isotopic compositions (food‐web perspective) or only using prey isotopic compositions (trophic niche scale). See Rigolet et al. (2015) for additional mathematical description.

#### 2.2.4 Diversity of prey and predator communities

A variety of diversity indices were calculated to investigate whether the diversity of prey available and of predators were similar in the three habitats. First, maximum cumulative species richness (SR_cum._) of prey taxa was calculated for each habitat to assess its relation with ABEC values. SR_cum._ equaled the number of species collected in three hauls of a given habitat, assuming that gear catchability was similar for all species and habitats. For the *Haploops* habitat, subsets of three of the six or seven hauls were randomly sampled (10,000 random selections) to allow relevant statistical comparison.

Three indices were calculated for predator diversity: cumulative species richness, Simpson diversity, and Pielou's measure of species evenness. To test for differences in indices of predator diversity among habitats, permutational multivariate analyses of variance (PERMANOVA) was performed for each bay (i.e., a [12 or 13 hauls × 3 diversity indices] matrix). Next, PERMANOVA was performed for each predator‐community matrix (with densities expressed as individuals/km^2^) of each bay (i.e., a [12 or 13 hauls × 30 species] matrix) to test for a habitat effect. Before running each PERMANOVA, we verified that the criterion of multivariate homogeneity of group dispersion was satisfied (Anderson & Walsh, 2013). 10,000 permutations were performed per analysis. Two factorial correspondence analyses (FCAs), based on the same community matrices, were performed to describe predator assemblages among habitats. However, only fish densities were used to calculate factorial axes; densities of invertebrate competitors were used as supplementary descriptors.

#### 2.2.5 Food‐web description based on stable isotopes

##### Isotope analysis

All fish and invertebrate (both prey and predator) species collected were sorted and kept frozen at a mean temperature of −20°C. At least three individuals of each species were analyzed. Small individuals were pooled to reach the minimum weight for stable‐isotope analysis. Samples of muscle tissue were used for fish and mega‐invertebrates, whereas the whole body (minus gut contents) was used for small invertebrates. After samples were rinsed with Milli‐Q water, species containing calcium carbonates were separated into two subsamples: one was acidified (in 10% HCl) to remove inorganic carbonates and the other served to identify nitrogen composition. Lipid content in the tissue samples was not corrected because it was considered sufficiently low. Isotopic compositions of δ^13^C and δ^15^N were obtained by the Stable Isotopes in Nature Laboratory at the University of New Brunswick (Canada) using an isotope‐ratio mass spectrometer (Finnigan Delta).

#### 2.2.6 Food‐web description

All stable‐isotope compositions of fish, invertebrates, and organic matter sources were graphically analyzed (on two‐dimensional isotopic δ‐spaces). A hierarchical ascendant clustering (HAC) restricted to a matrix of stable isotopes of benthic invertebrate taxa was performed for each habitat in each bay. Euclidean distances and Ward aggregation criterion were used. Clusters (hereafter, “groups”) were selected based on the highest relative loss of inertia (Appendix Table S1). HAC was performed only to compare the number of invertebrate groups. These groups were also used to assess potential differences in trophic positions (e.g., distinction between potential competitors or prey of fish species).

Isotopic functional indices (IFIs) were calculated to quantify food‐web properties and the (whole) community isotopic niche, as well as the optimal use of available resources in each habitat of each bay (Table 1). These indices are derived from biological traits‐based functional indices (e.g., Laliberté & Legendre, 2010; Mason, Mouillot, Lee, & Wilson, 2005; Villéger, Mason, & Mouillot, 2008) using morphological and biological measurements to assess potential changes in the structure and functioning of biological communities. Rigolet, Thiebaut, Brind'Amour, and Dubois (2015), for multispecies benthic assemblages, and Cucherousset & Villéger (2015), for fish communities, expanded the idea of community‐wide isotopic metrics originally described by Layman, Arrington, Montana, and Post (2007) using biomass to weight the isotopic composition of taxa. More details about the IFIs can be found in the supplemental material of Rigolet et al. (2015). All signatures (from sources to predators) were used for the IFIs calculation at the food‐web scale.

#### 2.2.7 Description of the trophic niche available for predators

After graphical analysis of the two‐dimensional isotopic δ‐spaces, IFIs were recalculated, this time using only the isotopic composition of potential prey based on HAC grouping. This helped to assess the trophic niche available to predators and their optimal use of resources. IFIs were calculated for each habitat in each bay. Results are presented only for the Bay of Concarneau because a previous study demonstrated that a threshold of about 20 species is required to provide accurate IFI values (Brind'Amour & Dubois, 2013). Data in the Bay of Vilaine did not reach that threshold.

We tested the significance of differences in IFIs between each pair of habitats using a two‐tailed permutation test: 10,000 permutations were performed on each matrix of δ^13^C–δ^15^N isotopic composition to obtain 10,000 sets of IFIs per habitat. Then, differences in the IFIs of the original matrices (i.e., without permutation) were compared to the distribution of the IFI differences obtained from the 10,000 permutations (Legendre & Legendre, 2012). Pseudo *p*‐values were calculated based on the proportion of permuted values falling outside the two extremes of the permuted distribution. Hypotheses were rejected using the Bonferroni correction with a significance threshold of 5% divided by the number of permutation tests.

#### 2.2.8 Prey contributions to predator diets

We used the R package SIAR (Stable Isotope Analysis in R; Parnell & Jackson, 2013) to estimate prey contributions to predator diets in each habitat in each bay. Use of mixed models provided a finite number of solutions, as the number of food sources (i.e., prey sources considered) per model equaled the number of elements analyzed (two stable isotopes, δ^13^C and δ^15^N) plus one (Parnell, Inger, Bearhop, & Jackson, 2010). Input data included (1) means and standard deviations of isotopic compositions of the prey (food sources); (2) replicates of predators’ isotopic compositions; and (3) literature‐based trophic enrichment factors (TEFs), which consider uncertainty in both prey and predator signatures (Parnell & Jackson, 2013).

Three global TEFs were tested to assess whether changes in the TEF used might affect results of the mixed models. The nitrogen‐isotope signature δ^15^N usually increases by 2.5‰–4.5‰ (mean = 3.4‰) from prey to predator (Darnaude, Salen‐Picard, Polunin, & Harmelin‐Vivien, 2004; Minagawa & Wada, 1984; Post, 2002), while δ^13^C usually increases by l‰–2‰ (De Niro & Epstein, 1978; Wada, Mizutani, & Minagawa, 1991). The three TEFs tested were (1) +l‰ in δ^13^C and +4‰ in δ^15^N (TEF1), (2) +2‰ in δ^13^C and +3‰ in δ^15^N (TEF2), and (3) +1.5‰ in δ^13^C and +3.4‰ in δ^15^N (TEF3) (Darnaude et al., 2004). As no difference appeared, the results presented are those obtained with TEF3.

To test predators’ preference for prey providing more energy, prey taxa were considered based on their ABECs. HAC was performed on a matrix of amount of energy (product of *E*_*i*_ × π_*i*_ × *R*_*i*_) and accessibility (*A*_*i*_; in columns) for each taxon (in rows), using Euclidean distances and a Ward aggregation criterion. Groups were selected based on the highest relative loss of inertia. From the species in each of these HAC‐based ABEC group (Appendix Table S1), means and standard deviations of δ^13^C and δ^15^N signatures were calculated for each bay.

Additionally, the predators (considered as “consumers” in SIAR package) were grouped according to their mobility, which resulted from behavioral (e.g., exploration vs. homing strategies) and physiological components (e.g., swimming ability). The groups were defined based on expert judgment and the literature (Le Pape & Cognez, 2016; White & Brown, 2013). As stable isotopic compositions integrate food consumed over 3–4 weeks (Vander Zanden, Clayton, Moody, Solomon, & Weidel, 2015), four groups were defined: species moving (1) ≤0.1 km; (2) 0.1–1 km; (3) 1–10 km; and (4) >10 km within this period (Appendix Table S2). All mega‐invertebrate predators were assumed to belong to the lowest mobility group.

Six distinct mixed models were used (one per habitat in each bay) because the data changed depending on the predators sampled in each habitat. Two datasets (one per bay) and one global TEF were used (i.e., TEF3). The prey contributing to the diets of predators—grouped by mobility—were analyzed for each habitat.

#### 2.2.9 Size‐spectra analyses of fish

To test the refuge‐area hypothesis, we searched for potential differences in the sizes of fish species in the habitats, using size‐spectra analyses. For each bay, the size spectrum of the fish community was calculated for the *Haploops* habitat and compared to that for *Sternaspis* and *Amphiura/Owenia* habitats combined (i.e., outside *Haploops* habitat), due to a dearth of data. The same approach was also used at the mobility‐group level, to assess whether the most mobile species behave like the least mobile species or, in contrast, seek higher energy prey in other habitats. Differences in the modes of size‐spectra distributions were assessed using a chi‐squared test. Finally, we analyzed which species contributed most to the mode of the community size spectrum.

All analyses were performed using R software R Core Team, 2015 and the packages as stated herein.

## 3 Results

Results from the multiple analyses are grouped and presented according to the three general hypotheses stated in the introduction: whether *H. nirae*, (1) modified the diversity, species composition, and biomass of invertebrates and fish juveniles; (2) affected food webs by creating preferential feeding grounds; and (3) created refuges for fish juveniles.

### 3.1 Prey and predator communities’ description

#### 3.1.1 Comparison of prey communities using the Available Benthic Energy Coefficients

The “ABEC × *B* ×*S*” values of *Haploops* habitat in the Bays of Concarneau and Vilaine were higher than those of the other two habitats in each bay (Table 2). *Haploops* habitat also had the largest area, highest maximum SR_cum_, and highest biomass. *Sternaspis* habitat had an *“*ABEC × *B* × *S*” value higher than that of *Amphiura/Owenia* habitat. The higher coefficient for *Sternaspis* habitat was associated with a larger area in the Bay of Vilaine only but with a higher biomass and a lower maximum SR_cum_ in both bays.

**Table 2:** Details of the components of Available Benthic Energy Coefficient (ABEC) calculated per habitat with the addition of habitat surface and maximal cumulated Species Richness (SR_cum._)

	Surface (*S*) (km^2^)	Average biomass (kg/km^2^)	ABEC × *B* (kJ·km^−2^·year^−1^)	ABEC × *B* × *S* (kJ·year^‐1^)	Cumulated (SR_cum._) species richness
Bay of concarneau					
*Amphiura/Owenia* habitat	68.7	8.4 × 10^2^	5.2 × 10^5^	3.6 × 10^6^	43
*Sternaspis* habitat	2.9	1.2 × 10^3^	4.5 × 10^6^	1.3 × 10^7^	35
*Haploops* habitat	81.1	2.3 × 10^4^	1.6 × 10^8^	1.3 × 10^10^	48 ± 3
Bay of Vilaine					
*Amphiura/Owenia* habitat	23.8	2.7 × 10^2^	8.1 × 10^5^	1.9 × 10^7^	32
*Sternaspis* habitat	96.3	7.4 × 10^2^	1.6 × 10^6^	1.6 × 10^8^	26
*Haploops* habitat	173.0	1.7 × 10^3^	2.4 × 10^6^	4.2 × 10^8^	33 ± 2

For *Haploops* habitat, subsets were randomly sampled among three on the six hauls, SR_cum._ means with standard deviation were calculated on 10,000 samplings.

### 3.2 Characteristics of predator communities

Predator‐community diversity indices showed significant differences in the cumulative species richness among the three habitats in the two bays (Table 3), with the highest diversity found in the *Haploops* and the *Amphiura*/*Owenia* habitats in the bay of Concarneau and Vilaine, respectively.

**Table 3:** Description of the predators’ communities and associated results from the statistical analyses. (A) Analyses based on predators’ diversity indices with separate PERMANOVAs performed for each bay. Post hoc tests were used to investigate the differences observed. (B) Analyses assessing potential differences in predators’ community abundance with separate PERMANOVAs performed for each bay

	*Amphiura/Owenia* habitat	*Haploops* habitat	*Sternaspis* habitat	Significance
A.				
PERMANOVA on diversity indices matrix (Bay of Concarneau)				a
Cumulative species richness	9.7	12.7	8.3	a
Simpson's diversity	0.6	0.5	0.6	n.s.
Pielou's eveness	0.9	0.7	0.7	n.s.
PERMANOVA on diversity indices matrix (Bay of Vilaine)				a
Cumulative species richness	15.7	9.3	12.7	a
Simpson's diversity	0.6	0.7	0.8	n.s.
Pielou's eveness	0.8	~1	~1	n.s.
B.				
PERMANOVA on predators community matrix (density data)				
Bay of Concarneau				a
Bay of Vilaine				n.s.

a A significance level α of .05 was used, and a Bonferroni method was used to adjust *p*‐values for post hoc tests.

**Figure 2:**
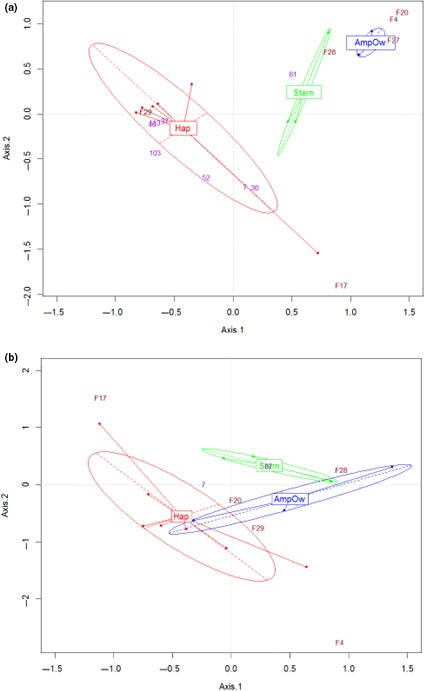
(a) Factorial correspondence analysis of fish community abundances for the Bay of Concarneau. (b) Factorial correspondence analysis of fish community abundances for the Bay of Vilaine. Both factorial maps display the sampling sites and the fish species (with a cosine^2^ > 0.4) with the addition of the ellipses of dispersion of each habitat. Megafaunal invertebrate species were considered as supplementary variables and superimposed to the factorial map color‐coded in violet and numbered according to Appendix Table S1

The community structure of the predators was significantly different among the three habitats in the Bay of Concarneau whereas no difference was found in the Bay of Vilaine (Figure 2 and Table 3). In the Bay of Concarneau, the FCA explained ~60% of the total inertia in the factorial map of the first and second axes (Figure 2a). The analysis separated groups of hauls from the three habitats, each of which associated with a different species assemblage. The *Haploops* habitat was associated mainly with a high density of *Trisopterus minutus* (poor cod, plotted near the barycenter of *Haploops* sampling sites/hauls) and *Merlangius merlangus* (whiting), which had a particularly high abundance in haul no. 11 (Figure 2a). The predator assemblage associated with *Haploops* habitat differed from those in the *Sternaspis* and *Amphiura/Owenia* habitats, which contained species such as *Trisopterus luscus, Pomatochistus minutus, Trachurus trachurus,* and flatfish (e.g., *Buglossidium luteum*). Some mega‐invertebrates were also more observed in *Haploops* habitat than in nearby habitats. It was the case for the polychaetes *Sthenelais boa* and *Eunice vittata*, the decapods *Inachus dorsettensis* and *L. depurator*, and the cephalopod *Allotheutis subulata*.

In the Bay of Vilaine, the FCA on the predator communities explained 44% of total inertia in the factorial map of the first and second axes (Figure 2b). Although the three habitats on the FCA can be distinguished (with some overlapping species), the PERMANOVA did not find any significant differences in the predator assemblages among the three habitats. Indeed, FCA in the Vilaine highlighted more common profiles of species density among habitats. However, some particularities were observed flatfish (e.g., *Solea solea, B. luteum*) were associated more with habitats outside *Haploops* habitat, whereas species such as *Zeus faber* and *Gobius niger* were associated more with *Haploops* habitat.

### 3.3 Haploops as potential feeding grounds

#### 3.3.1 Food‐web description based on stable isotopes

**Figure 3:**
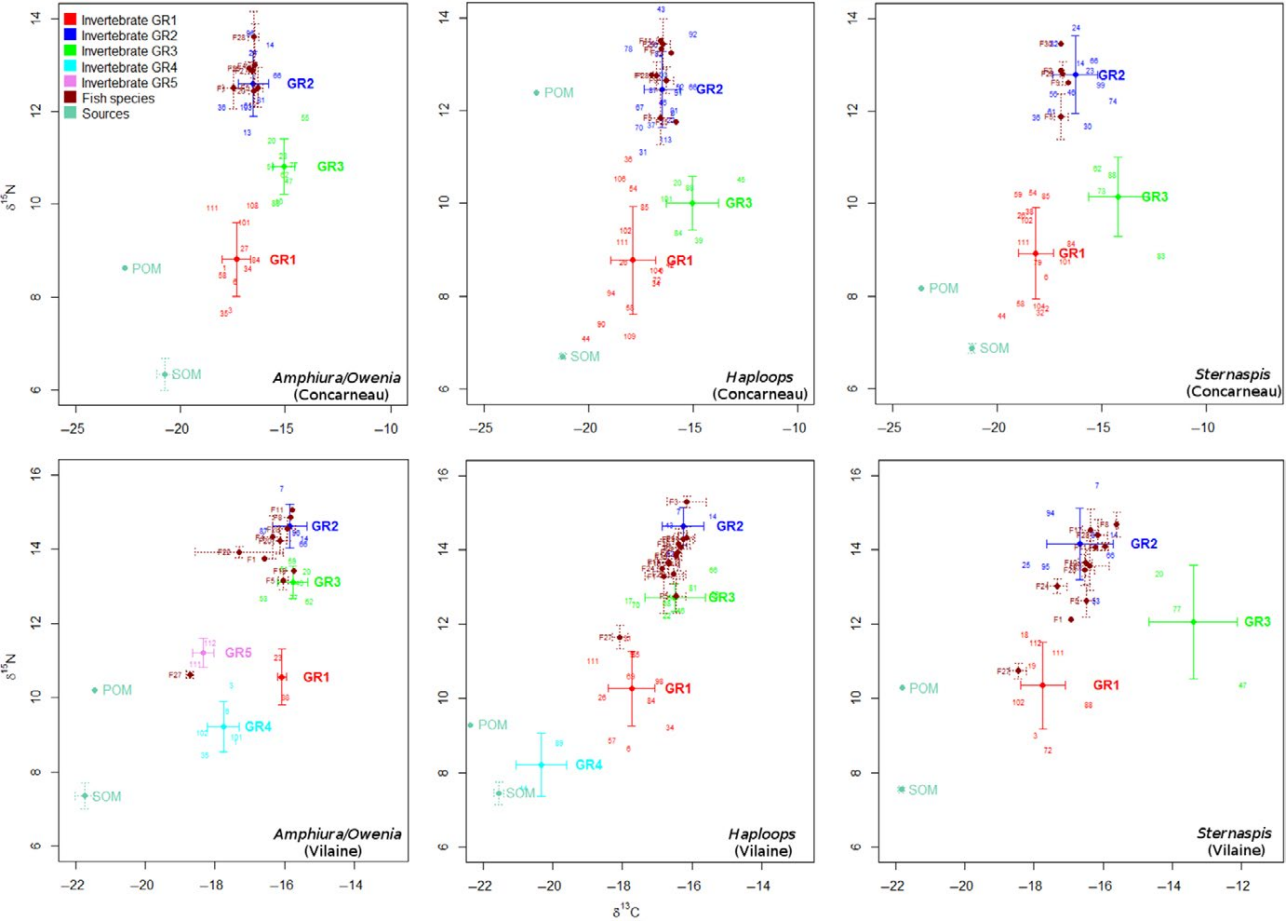
Two‐dimensional isotopic δ‐spaces (δ^15^N–δ^13^C) including the stable isotopic compositions of producers (organic matter sources), and first and second consumers (benthic mega‐invertebrates and fish). Species (labeled with numbers, see Appendix Table 1) were color‐coded according to the HAC grouping. All habitats for each bay were represented as specified in the top‐right corner of each biplot. It is worth noting that each bay has its own δ^13^C and δ^15^N scales

The cluster analysis (performed on isotopic compositions of invertebrate taxa) distinguished 3–5 groups per habitat. The Bay of Vilaine, despite having fewer species per habitat than the Bay of Concarneau (24–27 and 30–37, respectively), was composed of more groups in the *Amphiura*/*Owenia* habitat (5 and 3, respectively) and the *Haploops* habitat (4 and 3, respectively). Graphical analysis of HCA‐group projections on two‐dimensional isotopic δ‐spaces showed that some groups (GR2 in Concarneau, GR2 and GR3 in Vilaine; Figure 3) constitute potential competitors of fish species as their signatures overlapped those of fish species. These groups are composed mainly of omnivorous (such as the decapods *I. dorsettensis* [Jones et al., 1999] or *Necora puber* [McLuscky & Elliott, 2004]) and carnivorous (for instance the cephalopods *A. subulata* and *Sepia officinalis* or the polychaete *Aphrodita aculeata*) mega‐invertebrates that may share prey with fish.

Comparison of the isotopic diversity indices, using a two‐tailed permutation test, underlined significant differences in the range of the δ^15^N (NR) with the *Haploops* habitat having a higher NR than the other habitats in the Bay of Vilaine (Table 4). A significant difference in NR was also observed between *Haploops* and *Sternaspis* habitats in the Bay of Concarneau. No significant difference was observed in values of the SEA, a proxy of isotopic richness (or isotopic niche width), in either bay. In contrast, functional divergence (IDiv) was significantly lower in the *Amphiura/Owenia* habitat in the two bays.

**Table 4:** Isotopic functional indices computed on the food webs of each habitat (see isotopic functional indice codes in Table 1)

	Bay of Concarneau			Bay of Vilaine		
	*Amphiura/Owenia* (A/O) habitat	*Sternaspis* (S) habitat	*Haploops* (H) habitat	*Amphiura/Owenia* (A/O) habitat	*Sternaspis* (S) habitat	*Haploops* (H) habitat
SEA	5.30	9.74	7.54	5.58	5.05	3.86
NR	6.04	*6.22*	*7.04*	7.17	6.05	**7.68** *
IDiv	**0.49** *	0.54	0.64	*0.54*	0.88	*0.99*
*n*	30	30	37	27	24	26

Based on two‐tailed permutation tests, differences between a given habitat and the two others are indicated in bold, and italics specifies when a difference is only observed between the two underlined habitats. *A significance level α of .05 was used.

#### 3.3.2 Description of the trophic niche available for predators

As mentioned in the Section 2, only the results for the Bay of Concarneau is showed as we did not reach the required minimum sample size in the Bay of Vilaine to correctly perform the analysis. Therefore, the comparison of the trophic niche available to predators in the Bay of Concarneau showed a clear distinction among habitats (Table 5). SEA was significantly lower in *Amphiura/Owenia* habitat than in the other two habitats, which were not significantly different from each other. NR in *Sternaspis* habitat was significantly lower than those in *Haploops* or *Amphiura/Owenia* habitats. IDiv distinguished *Haploops* habitat from the other two habitats, whereas functional dispersion (IDis) in *Amphiura/Owenia* habitat was significantly higher than those in *Haploops* or *Sternaspis* habitats. Finally, no significant differences were observed in isotopic eveness (IEve) values.

**Table 5:** Isotopic functional indices estimated on the prey and characterizing the isotopic niche available for the predators of the Bay of Concarneau (see isotopic functional indice codes in Table 1)

	Indices values			Pseudo *p*‐values		
	*Amphiura/Owenia* (A/O) habitat	*Sternaspis* (S) habitat	*Haploops* (H) habitat	H—A/O	H—S	A/O—S
SEA	2.41	6.63	5.89	**<.001**	.50	**<.001**
NR	4.22	3.18	3.80	.99	**<.001**	**<.001**
IDiv	0.64	0.64	0.39	**<.001**	**.002**	.99
IDis	1.17	0.62	0.84	**<.001**	.06	**<.001**
IEve	0.30	0.52	0.24	.73	.37	.87

The pseudo *p*‐values from the comparisons between each pairs of habitat of two‐tailed permutation tests are also given. A significance level α of .05 was used.

### 3.4 Food‐source contributions to the predator diet

Results from the three habitats showed very similar patterns, therefore only those for *Haploops* habitats are presented here. However, the complete results including those of *Haploops* can be found in Appendix Fig. S1.

The cluster analysis conducted on the species ABEC values distinguished three groups of prey (result not showed). These groups were first distinguished based on their amount of energy, with ABEC group 1, group 2, and group 3 having lower, intermediate, and higher energy, respectively. The groups were secondly segregated by their accessibility; with ABEC group 1 composed of accessible taxa whereas ABEC group 2 and group 3 with less‐accessible taxa (see Tableau et al., 2015 for accessibility details).

**Figure 4:**
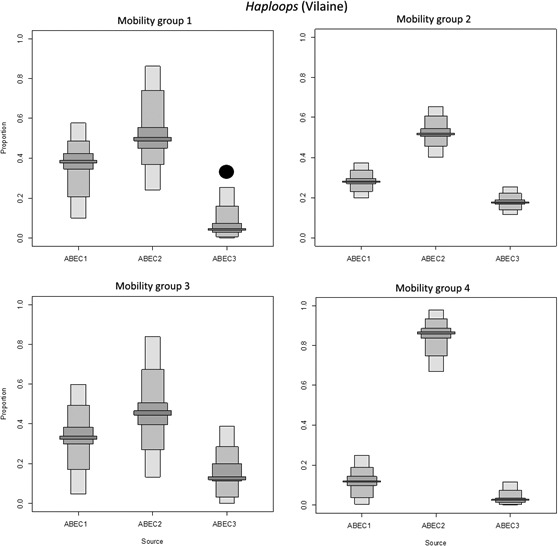
Contribution of the Available Benthic Energy Coefficient (ABEC) groups to the diet of predators (fish and invertebrates) in the *Haploops* habitat of the Bay of Vilaine using SIAR mixing model. Box plots illustrate the 25th, 50th, and 75th percentiles; the whiskers indicate the 10th and 90th percentiles. The details of ABEC‐based sources’ compositions (i.e., ABEC groups 1–3) are given in the Appendix Table S1. Top left to bottom right panels display the results for mobility group 1 (up to 100 m), mobility group 2 (between 100 m and 1 km), mobility group 3 (between 1 and 10 km), and mobility group 4 (above 10 km). The black circle indicates the ABEC source associated with the highest accessible biomass, and the black square indicates the source with the highest energetic supply (see Section 2 for complementary information)

**Figure 5:**
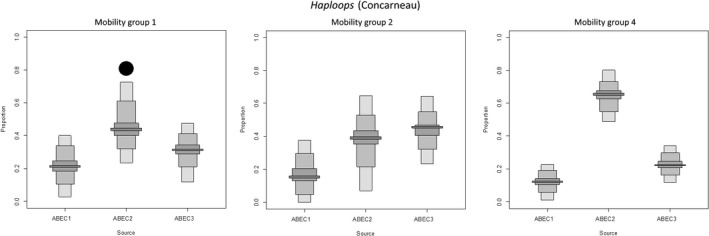
Contribution of the Available Benthic Energy Coefficient (ABEC) groups to the diets of predators in the *Haploops* habitat of the Bay of Concarneau, using SIAR mixing model. Box plots illustrate the 25th, 50th, and 75th percentiles; the whiskers indicate the 10th and 90th percentiles. The details of ABEC‐based sources’ compositions (i.e., ABEC groups 1–3) are given in the Appendix Table S1. From top left to bottom are displayed the results for mobility group 1 (up to 100 m), mobility group 2 (between 100 m and 1 km), and mobility group 4 (above 10 km). Due to a limited number of data to perform the analysis of mobility group 3, result was not presented here. The black circle indicates the ABEC source associated with the highest accessible biomass, and the black square indicates the source with the highest energetic supply (*E*_*i*_ × π_*i*_ × *R*_*i*_)

Comparisons in predators’ diets using SIAR showed two distinct patterns. First, among the three sources, the ABEC group 2 (composed of less‐accessible prey with an intermediate amount of energy) contributed most to the predator diet, regardless of the bay, habitat, or mobility group. This pattern was found in 78% of the analyses conducted. Second, in 22% of the analyses, a (co‐preference or a) slightly higher contribution of ABEC group 1 or 3 was observed (Figures 4, 5 and S1).

In accordance with the two patterns determined above, the habitats from the two bays were classified in two groups:

1. The first group was composed of *Haploops* habitat in the Bay of Vilaine (Figure 4) and *Amphiura/Owenia* habitat in the Bay of Concarneau (i.e., two of the six habitats). In those two habitats, the source composed of ABEC group 2 taxa was found in highest proportion in the diet of the four mobility classes of predators but was not the most accessible or highest energy prey. Indeed, the most energetic having the higher available biomass in the *Haploops* habitat in the Bay of Vilaine, was the ABEC group 3, composed mainly of *H*. *nirae*. Concurrently, in the *Amphiura/Owenia* habitat in the Bay of Concarneau, it was the ABEC group 1 who had the most accessible and highest energy biomass, due to *A. filiformis*.
2. The second group of habitats was composed of four of the six habitats, including the *Haploops* habitat in the Bay of Concarneau. In that group, beside the exception of possible co‐preference (e.g., the mobility group 2 of *Haploops* habitat in the Bay of Concarneau), the ABEC group 2 was the main contributor to the predators’ diets and had the most accessible biomass and the highest amount of energy (Figure 5).

### 3.5 Potential refuges for fish juveniles

**Figure 6:**
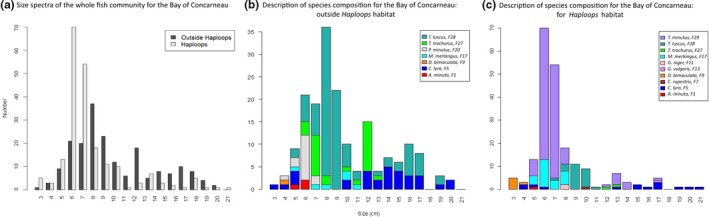
Size spectra of the whole fish community in *Haploops* habitat (in white), and outside *Haploops* habitats together (in black) (a) For the Bay of Concarneau. Description of species composition for the Bay of Concarneau: (b) Outside *Haploops*. (c) For *Haploops* habitat

The refuge hypothesis was mainly addressed by the size‐spectrum analyses. In the Bay of Concarneau, the modes of size distribution were significantly different (*p* < .05) between *Haploops* habitat (6 cm) and surrounding habitats (8 cm) (Figure 6a). In the Bay of Vilaine, however, habitats had no significant differences in mode (all centered on 8 cm) (result not showed). The difference in modes in the Bay of Concarneau was due mainly to *T*. *minutus* in *Haploops* habitat, which was replaced by *T. luscus* in nearby habitats (Figure 6b,c). Other main contributors to the difference in modes were *M. merlangus* in *Haploops* habitat (even though some *M. merlangus* individuals were observed also outside that habitat) and *T. trachurus* outside *Haploops* habitat.

Additional size‐spectrum analyses were conducted at the scale of the mobility groups. They were carried exclusively on the fish mobility groups 3 (in the Bay of Concarneau) and 4 (in the two bays), as only those group had sufficient data (Appendix Fig. S2). The mobility group 3 was composed primarily of flatfish, only observed in the Bay of Concarneau (Appendix Table S1), which were however absent (exclusion pattern) from *Haploops* habitat (Fig. S2A). Size spectra of mobility group 4 differed significantly among habitats, showing smaller modes in the *Haploops* habitat in the Bay of Concarneau in comparison with other habitats (*p* < .05). This result was coherent with the significant difference in size spectra of the entire fish community, as the community was mainly composed of species from mobility group 4 (Fig. S2B). In contrast, no difference was observed in size spectra of mobility group 4 among habitats in the Bay of Vilaine (Fig. S2C).

## 4 Discussion

This study investigated functional roles of engineered habitats, using the particular case of *H. nirae* and the habitat it creates in shallow marine water. This was done by assessing three main consequences of species engineering on its habitat: (1) effects on associated species composition and diversity, (2) creation of feeding opportunities, and (3) potential refuges. Guided by these aspects and the framework of Jones et al. (1994), we present a conceptual model of *H. nirae* as an engineer species.

### 4.1 Influence of *Haploops nirae* on benthic invertebrate and fish diversity and biomass

Our results highlighted a higher cumulative species richness and energetic value of benthic invertebrates in *Haploops* habitat than in the two other habitats, in both bays. This higher diversity was associated with higher biomass of benthic invertebrates. Rigolet, Dubois, and Thiébaut (2014) emphasized the uniqueness of this habitat's macro‐fauna species assemblages, with one‐third of all macro‐invertebrate species recorded in the Bay of Concarneau found exclusively in *Haploops* habitat. They also found that species richness in *Haploops* habitat was significantly higher than that in nearby sediments not colonized by *Haploops*. Similar observations were made about macro‐fauna assemblages in the Bay of Vilaine (Le Bris & Glémarec, 1995). The higher species richness was also found in mega‐invertebrate communities in the bays. Because *Haploops* habitat occupied the largest area and had the largest mean (prey) biomass, its energetic value—estimated *via* ABEC—was also the highest among the three habitats in the two bays.

Comparisons of predator (i.e., fish and megafaunal invertebrates) communities among the habitats in the bays identified differences in species richness and community structure. Differences in predator assemblages were identified for the Bay of Concarneau (only partially for the Bay of Vilaine) in *Haploops* habitat and outside *Haploops* habitat. Differences in fish assemblages in *Haploops* habitat in the Bay of Vilaine were previously documented with a characterization of fish groups belonging to (1) ubiquitous or constant species (i.e., equally distributed in both habitats), (2) preferring species (i.e., found in one habitat or the other), and (3) avoidant species (i.e., rare or absent in a given habitat; Désaunay, Laffargue, & Lobry, pers. comm.). These authors distinguished preferring species found in *Haploops* habitat (e.g., *Scyliorhinus canicula*,* Trisopterus* spp., *Labrus bergylta*,* Zeus faber*) from flatfish (e.g., *S. solea, Pleuronectes platessa)* found exclusively outside *Haploops* habitat. Our results do not entirely support Désaunay, Laffargue, & Lobry (pers. comm.), as, among *Trisopterus* spp. in the Bay of Concarneau, *T. minutus* was found more often in *Haploops* habitat, while *T. luscus* was found more often outside *Haploops* habitat. One persistent pattern, however, is the exclusion of flatfish from *Haploops* habitats in the two bays. This agrees with the literature dedicated to flatfish, such as that for *S. solea* (Kopp, Le Bris, Grimaud, Nerot, & Brind'Amour, 2013; Le Pape et al., 2013).

Our results agree with the general trend usually observed for engineered habitats: an increase in species richness and changes in abundance resulting from an increase in habitat heterogeneity/complexity, both leading to modified species assemblages (Castilla et al., 2004; Crooks & Khim, 1999; Thomas et al., 1998; Wright et al., 2002). In their review, Gutiérrez et al. (2011) presented marine coastal examples of physically engineered habitats involving structural change in the environment, such as the creation of depressed roughness elements (e.g., burrows, depressions caused by crustaceans) of variable persistence or emergent massive structures (e.g., mangroves, seagrass, coral, or bivalve reef constructions), that increase species richness of associated assemblages. Opposite trends can also be found in the literature, especially for invasive engineer species. For example, rapid proliferation of *Crepidula fornicata* (American slipper‐limpet) in Atlantic nursery grounds in France considerably decreased juvenile sole density by decreasing the area available for flatfish (Kostecki et al., 2011). Noninvasive engineer species, however, may also decrease species richness. Tube‐building polychaetes generally need stable sediments to establish and develop, but bioturbation destabilizes sediments (Brenchley, 1981). Consequently, an expanding bioturbator community may lead to exclusion of tube‐building polychaetes. Our results of distinct predator assemblages raise the question of whether the habitats have functional differences, serving as preferential feeding grounds (due to more abundant, more diverse and/or higher energy prey) or refuge areas. Ultimately, do these functional changes impact food‐web structure at the habitat scale?

### 4.2 *Haploops* habitat as a preferential feeding area

IFIs were used to investigate the valuable feeding ground hypothesis. They assume that isotopic space is a proxy of an organism's trophic niche, integrating information about its physical habitat (e.g., hydro‐climatic conditions) and trophic characteristics (Bearhop, Adams, Waldron, Fuller, & MacLeod, 2004; Dubois & Colombo, 2014; Newsome, Martınez del Rio, Bearhop, & Phillips, 2007). These indices, initially applied to populations, have also been applied to communities, revealing food‐web properties and functioning characteristics (i.e., diversity, redundancy), even though some caveats have to be considered (Hoenghaus & Zeug, 2008; Layman et al., 2007). Recent studies have shown the relevance and necessity of weighting isotopic compositions by species biomass to further explore food‐web properties (Cucherousset & Villéger, 2015; Rigolet et al., 2015).

Comparison of IFIs calculated to describe the possible differences in food webs supported the graphical analysis of two‐dimensional δ^13^C‐δ^15^N spaces. A wider range in δ^15^N signatures was observed in *Haploops* habitat in both bays, suggesting longer trophic chains. No difference in SEA was observed among habitats in either bay, suggesting that they all had similar levels of trophic richness. The isotopic functional divergence (IDiv) was high in *Haploops* habitats, especially compared to *Amphiura/Owenia* habitats (the lowest IDiv in both bays), suggesting that a higher proportion of the biomass in *Haploops* habitat originated from alternative (i.e., micro‐phytobenthic) food sources. This result is supported by previous studies that demonstrated high benthic primary production in *Haploops* habitat and the contribution of this food source in ecosystems that *H. nirae* colonizes (Kopp et al., 2013; Rigolet et al., 2014). It is also a general feature of intertidal communities in engineered habitats (Passarelli, Olivier, Paterson, & Hubas, 2012; Passarelli, Olivier, Paterson, Méziane, & Hubas, 2014).

The potential of *Haploops* habitat to serve as a preferential feeding ground was also analyzed through the size of the trophic niche available for predators, that is, based on isotopic signatures of invertebrate prey only. This time, the comparison of functional indices identified a higher SEA and lower isotopic dispersion (IDis) for *Haploops* and *Sternaspis* habitats in comparison with the third habitat. This is suggesting, for instance, that the prey in *Haploops* habitat are offering a larger trophic niche dominated by few generalist species (i.e., species closer to the center of gravity). Concurrently, the lower IDiv of *Haploops* habitat may indicate that one main trophic pathway dominated, with *H. nirae* at its base, as observed by Rigolet (2013). This result also agrees with that of Mackenzie et al. (2006), who showed that *Haploops* facilitated suspension‐feeders development. Interestingly, this result was not consistent with that of Rigolet et al., 2015, whose IFIs (including IDiv and IDis) identified more trophic specialization and niche differentiation in *Haploops* habitat than in *Amphiura/Owenia* habitat; however, they considered the entire invertebrate community, including predators.

The use of IFIs at the food‐web versus prey level may fully change understanding of food‐web properties and lead to opposing conclusions. In our case, focusing only on the prey provided additional insights about profitable resources and the available trophic niche.

Despite *Haploops* habitat's higher functional richness (than *Amphiura/Owenia* habitat), trophic chain length (than *Sternaspis* habitat) and higher IDis, its main characteristic was its low IDiv. Its lower IDiv was related to its lower specialization, which also agreed with the lower—but not significantly different—functional evenness. This is another proxy of trophic redundancy. Even though the prey widened the trophic niche in *Haploops* habitat, other properties seem to reflect a habitat homogeneity which is more likely to offer less resistance to disturbances or stress (Rigolet et al., 2015). Complementary information was provided by the use of mixed models. Our results suggested that preference for prey varied according to the predators considered (i.e., mobility groups) and that it was also highly sensitive to the sample size. The feeding preference was clearer when the “consumers” data were at least twice the size of the sources data considered in the mixed models (in our case at least 2 × 3; that is, at least six consumers’ data). In most cases, the source of prey preferred had the most accessible biomass and the highest amount of energy. This may indicate an opportunistic strategy when species only seek highly accessible biomass; the convergence with an energetic interest precluded testing for energy optimization in the foraging strategy. However, highly accessible biomass or high energetic interest did not always determine the source of preferred prey, such as in *Amphiura/Owenia* habitat in the Bay of Concarneau and *Haploops* habitat in the Bay of Vilaine. In each of the two habitats, the dominant species (*A. filiformis* and *H. nirae*, respectively) was the source with the highest accessible biomass and amount of energy, but they are not belonging to the group of preferred prey. These results partly demonstrate that predators’ strategies are not entirely opportunistic, contrary to what is often discussed in the literature (Laffaille, Lefeuvre, Schricke, & Feunteun, 2001; Maes & Ollevier, 2002; Roberts, Xavier, & Agnew, 2011; Sá, Bexiga, Veiga, Vieira, & Erzini, 2006). Likewise, in the Canche Estuary nursery (France), differences between available prey and fish diets were described, suggesting that fish do not always select the most abundant food resources (Selleslagh & Amara, 2015). Nor, according to our results, do they always select the prey with the most energy. Our results indicate the need to further improve indicators such as ABEC by adding information to refine results such as prey digestibility, which may affect available energy content (Hajen, Higgs, Beames, & Dosanjh, 1993; Lindsay et al., 1984). Another possibility is to consider prey size in more detail. Barry, Yoklavich, Cailliet, Ambrose, and Antrim (1996) and Gning, Vidy, and Thiaw (2008) noted that fish prefer larger prey, which are also more energy‐rich, as they grow. Similarly, Selleslagh and Amara (2015) showed that increased mouth size allows fish to consume higher energy prey, such as polychaetes. Although size was considered by excluding prey species larger than the largest fish mouth (Tableau et al., 2015; Wainwright & Richard, 1995) from the list of species for which ABEC was calculated, energy indices such as ABEC likely require some methodological improvements.

### 4.3 *Haploops* habitat as a potential refuge

Size‐spectra analysis was used to investigate the potential of *Haploops* habitat as a refuge for juvenile fish. Our results indicated a significant decrease in the size distribution of the fish community in the Bay of Concarneau (mode from 8 to 6 cm), indicating the presence of smaller individuals in *Haploops* habitat. This result was also confirmed by the size spectra of two mobility groups with the highest mobility (i.e., between 1 and 10 km and >10 km in 3–4 weeks), while the individuals sampled in *Haploops* habitat could move to other habitats. The species associated with the modes of distributions highlighted a clear change in the main contributor species (*T. luscus* outside *Haploops* habitat and *T. minutus* in *Haploops* habitat), a change that drove the change in size spectra. As these species are well known to differ in size, this result was expected (and also observed on the assemblages described by FCA). *T. minutus* feeds mainly on epibenthic and benthopelagic invertebrates, such as polychaetes, decapods, and amphipods (Mattiangeli, Bourke, Ryan, Mork, & Cross, 2000; Mattson, 1990). The size and variety of its prey appear related to its size (Armstrong, 1982; Mattiangeli et al., 2000), and no clear change has been observed in the quality of food it may ingest during a year (Politou & Papaconstantinou, 1994). For *T. luscus*, seasonal feeding activity*,* which may influence its prey preference, has yet to be investigated (Alonso‐Fernández & Saborido‐Rey, 2012). The question remains whether these differences are substantive as their feeding preferences may change or their use of a habitat as a refuge may influence their diet. Finally, size‐spectra analysis of the group with mobility of 1–10 km in 3–4 weeks (i.e., flatfish) demonstrated their clear exclusion from *Haploops* habitat, in line with previous observations.

Fish size was the only explanatory variable used to test the refuge‐area hypothesis. Despite limitations of our approach, size differences may reflect differences in individuals’ growth rates and, consequently, health (although these factors may not fluctuate in parallel; Sinovčid, Keč, & Zorica, 2008). This idea is based on the assumption that isometric growth is a fair approximation of growth for many fish species (Jones, Petrell, & Pauly, 1999; Kimmerer et al., 2005). Some conflicting results seem to invalidate this argument, however. First, ABECs calculated for each habitat suggest that *Haploops* habitat may offer more prey biomass, diversity, and energy than other nearby habitats. Predators, especially fish species with feeding strategies besides opportunistic, should not encounter fewer resources in *Haploops* habitat. Fulton's (1904) index, widely used in fish biology describes the relation between fish weight and length and serves as an indicator of fish health. Calculation of Fulton's index for the studied habitats revealed similar values for each bay (results not shown), confirming that poor health did not drive the smaller modal size of fish in *Haploops* habitat. Based on the density of *H. nirae* individuals (i.e., 2,500–10,000 tubes (individuals)/m^2^; Rigolet, 2013), we estimated a spacing of 1–2 cm between *Haploops* tubes. This distance seemed compatible with the estimated width and the hiding behavior (between *H. nirae* tubes) of certain species (e.g., *T. minutus*; Reecht, Rochet, Trenkel, Jennings, & Pinnegar, 2013), which also has been observed in diving photographs (X. Caisey, IFREMER, pers. comm.).

### 4.4 Understanding the Haploops habitat as an engineered habitat

Referring to reviews by Jones et al. (1994) and Lawton (1994), we verified four classic characteristics of engineered habitats:

C1 Engineers create, modify, or maintain habitats, and consequently affect local environmental conditions and resource availability (Jones et al., 1994).
C2 By increasing spatial heterogeneity, engineers modify (local) species diversity (Crooks, 2002; Hendy et al., 2014; Jones et al., 1997; Wright & Jones, 2006) *via* changes in species richness or abundance.
C3 These changes may influence species interactions, trophic niche differentiation, trophic levels (Crain & Bertness, 2006; Erwin, 2005), and food‐web functioning (Sanders et al., 2014).
C4 Engineered habitats, like other highly structured habitats, can provide refuge areas for certain species (Hastings et al., 2007; Hendy et al., 2014).

**Figure 7:**
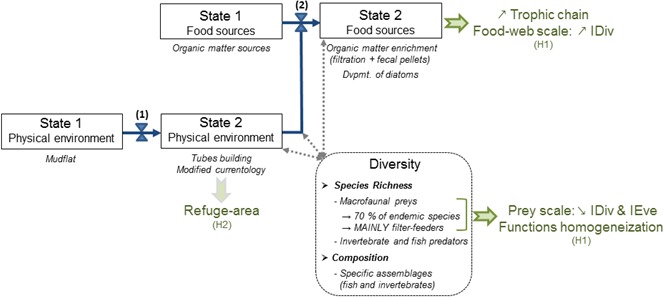
Diagram synthesizing the functional roles of the ecosystem engineer *Haploops nirae* based on the results found in this study (*in italics*) and from Rigolet (2013). The scheme is adapted from Jones et al. (1994). (1) indicates the direct alteration of the physical environment via tubes building. (2) indicates the modification of organic matter sources (filtering activity and fecal pellets production). The two effects have consequences in terms of diversity of invertebrates and fish species inhabiting (temporarily or not) *Haploops* habitats. They also result in functional changes (in green). H1: the hypothesis on the preferential feeding ground. H2: hypothesis on the refuge area

We verified these characteristics in our case study of engineered habitat and compared them to our results in a summary diagram (Figure 7).

*Haploops nirae* engineering activity can be a combination of two allogenic processes (Jones et al., 1994). First, its tube building may be regarded as an increase in sediment heterogeneity, changing a site from a mudflat to patches of amphipod tubes (Ehrhold et al., 2006; Glémarec et al., 1986; Rigolet, 2013). In the physical environment, addition of *H. nirae* tubes may also change local granulometry by influencing boundary*‐*layer flows (C1; Friedrichs, Graf, & Springer, 2000). Second, production of feces and pseudofeces by *H. nirae* modifies organic matter sources, which offers a substrate suitable for enhancing development of micro‐phythobenthos (C1; Rigolet et al., 2014).

Consequences on the diversity of invertebrates and fish species inhabiting *Haploops* habitats were also highlighted (C2). The higher diversity of invertebrates was consistent with previous studies, as did the 70% of endemic species and 33% of unique species being exclusively associated with *Haploops* habitat (Rigolet et al., 2013; our study). Increased predator diversity was also found in *Haploops* habitat in the Bay of Concarneau, although the opposite was found in the Bay of Vilaine, mainly related to the exclusion of flatfish species (our study). These differences were also confirmed by differing species assemblages when comparing species abundances among habitats (C2).

Functional changes were assessed while testing the preferential feeding ground hypothesis in *Haploops* habitat. In accordance with the literature, we observed longer trophic chains in *Haploops* habitat, partly supporting an increase in the trophic niche of the community (potentially related to more trophic levels, but only observed in the Bay of Vilaine) and in agreement with observations by Passarelli et al. (2012). Analyses also support higher isotopic divergence in *Haploops* habitat at the food‐web level due to the presence of an alternative food source (i.e., micro‐phytobenthos). Low isotopic divergence and evenness were observed, which supports results of Rigolet et al. (2015) of higher trophic specialization and niche differentiation (related to homogenization of trophic function) in *Haploops* habitat than in *Amphiura/Owenia* habitat (C3).

While the preferential feeding ground hypothesis was not supported, the refuge‐area hypothesis, another feature of engineered habitats (C4), seemed to be supported, but only for a few species (Figure 7).

Finally, the role of *H. nirae* as engineer species was underlined through the analysis of three main hypotheses (i.e., communities’ structure alterations, feeding ground, or refuge for species) and discussed above. The main remaining issue that could not be apprehended in this study is about the generalization of our observations. Indeed, based on the sampling performed, the two bays could not be directly compared, as their environmental conditions differ (salinity and depth gradient notably) and also the location and distance between the three studied habitats. Based on this fact, no direct comparison was also feasible and we do not know how these factors may influence our results. The directly related question or perspective to be investigated should concern the potential effect of temporal variability (interannual or seasonal), which could also change our understanding of this engineered habitat.

## Conflict of Interest

None declared.

## Data Accessibility

Densities and isotopic data are accessible from the publication link: Chaalali, Brind'Amour, Dubois, and Le Bris (2016). R scripts for IFIs calculation are made available by Sébastien Villéger (http://villeger.sebastien.free.fr/R%20scripts/FDchange.r), scripts for FCA and for PERMANOVA from ade4 package by S. Dray (https://pbil.univ-lyon1.fr/CRAN/web/packages/ade4/index.html) and vegan package by J. Oksanen (https://cran.r-project.org/web/packages/vegan/index.html), respectively, and SIAR by Andrew Jackson (www.cran.r-project.org/web/packages/siar/siar.pdf).
