# Supplementary material for: Functional roles of an engineer species for coastal benthic invertebrates and demersal fish
Source: Ecol Evol. 2017 Jun 15;7(15):5542–59. doi: 10.1002/ece3.2857 (PMC5552942; doi:10.1002/ece3.2857)
Supplement: Supplementary file 1 [file ECE3-7-5542-s001.doc]

**APPENDICES**

**Table S1**: Full description of invertebrate taxa considered in this study, including: species codes (used for example in two-dimentional δ-spaces), scientific names, ABEC details of calculation (E for mass energy in kJ.g^−1^; π the productivity in kJ.g^−1^.y^−1^; a coefficient of regeneration (R); a coefficient of accessibility (A), both unitless; and the average biomass in g.1000 m^-2^), respective ABEC groups, and HAC-based groups (basing on their isotopic signatures). Additional codes were used for each habitat: ‘Amp.Ow.’ for *Amphiura/Owenia* habitat, ‘Hap.’ for *Haploops* habitat, and ‘Stern.’ for *Sternaspis* habitat. ‘.CO’ for the Bay of Concarneau and ‘.VI’ for the Bay of Vilaine.

| **Species ID** | **Species scientific names** | **ABEC** | | | | | **Average biomass** (for habitat ABEC calculation) | | | | | | **ABEC-based group** | **HAC-based group** | | | | | |
| --- | --- | --- | --- | --- | --- | --- | --- | --- | --- | --- | --- | --- | --- | --- | --- | --- | --- | --- | --- |
|  |  | **E** | **π** | **R** | **E.π.R** | **A** | **Amp.Ow.CO** | **Hap.CO** | **Stern.CO** | **Amp.Ow.VI** | **Hap.VI** | **Stern.VI** |  | **Amp.Ow.CO** | **Hap.CO** | **Stern.CO** | **Amp.Ow.VI** | **Hap.VI** | **Stern.VI** |
| 1 | *Abra alba* | 1,11 | 1,22 | 1,50 | 2,03 | 0,11 | 6,32 | 0,00 | 0,00 | 1,77 | 0,00 | 0,00 | 1 | 1 | . | . | 1 | . | . |
| 2 | *Acanthocardia aculeata* | . | . | . | . | . | . | . | . | . | . | . | . | . | . | 1 | . | . | . |
| 3 | *Acanthocardia echinata* | 2,22 | 0,99 | 1,00 | 2,19 | 1,00 | 6,53 | 0,00 | 0,00 | 0,12 | 0,00 | 197,50 | 1 | 1 | . | . | 1 | . | 1 |
| 4 | *Acteon tornatilis* | . | . | . | . | . | . | . | . | . | . | . | . | . | . | . | . | . | . |
| 5 | Actinaria | . | . | . | . | . | . | . | . | . | . | . | . | . | . | . | . | . | . |
| 6 | *Aequipecten opercularis* | . | . | . | . | . | . | . | . | . | . | . | . | 1 | 1 | 1 | 1 | 1 | . |
| 7 | *Alloteuthis subulata* | . | . | . | . | . | . | . | . | . | . | . | . | . | . | . | 2 | 2 | 2 |
| 8 | *Alpheus macrocheles* | . | . | . | . | . | . | . | . | . | . | . | . | . | 2 | . | . | . | . |
| 9 | Amphitrite sp. | . | . | . | . | . | . | . | . | . | . | . | . | . | . | . | . | 1 | . |
| 10 | *Acrocnida brachiata* | 2,99 | 0,43 | 1,15 | 1,47 | 0,11 | 523,70 | 0,00 | 0,45 | 0,00 | 0,00 | 0,22 | 1 | 3 | . | . | . | . | . |
| 11 | *Amphiura filiformis* | 2,99 | 0,72 | 1,15 | 2,49 | 0,11 | 0,00 | 0,00 | 7,24 | 0,00 | 0,00 | 0,00 | 1 | . | . | . | . | . | . |
| 12 | *Anomia ephippium* | . | . | . | . | . | . | . | . | . | . | . | . | . | . | . | . | . | . |
| 13 | *Antalis vulgaris* | . | . | . | . | . | . | . | . | . | . | . | . | 2 | . | . | . | . | . |
| 14 | *Aphrodita aculeata* | 2,55 | 2,49 | 1,20 | 7,62 | 0,11 | 80,03 | 78,84 | 66,49 | 46,07 | 1,73 | 13,96 | 2 | 2 | . | 2 | 2 | 2 | 2 |
| 15 | *Aplysia punctata* | . | . | . | . | . | . | . | . | . | . | . | . | . | . | . | . | . | . |
| 16 | *Aporrhais serresianus* | . | . | . | . | . | . | . | . | . | . | . | . | . | . | . | . | . | . |
| 17 | *Aricia latreillii* | . | . | . | . | . | . | . | . | . | . | . | . | . | . | . | . | 3 | . |
| 18 | *Armina loveni* | . | . | . | . | . | . | . | . | . | . | . | . | . | . | . | . | . | 1 |
| 19 | *Asthenognathus atlanticus* | 3,76 | 2,14 | 1,00 | 8,04 | 0,11 | 0,00 | 0,00 | 0,00 | 0,00 | 0,00 | 0,67 | 2 | . | . | . | . | . | 1 |
| 20 | *Asterias rubens* | . | . | . | . | . | . | . | . | . | . | . | . | 3 | 3 | . | 3 | . | 3 |
| 21 | *Astropecten irregularis irregularis* | . | . | . | . | . | . | . | . | . | . | . | . | . | . | . | . | . | . |
| 22 | *Atelecyclus rotundatus* | 3,76 | 4,29 | 1,00 | 16,13 | 1,00 | 0,00 | 1528,05 | 0,00 | 0,00 | 6,96 | 0,00 | 3 | . | 2 | . | . | 3 | . |
| 23 | *Buccinum undatum* | . | . | . | . | . | . | . | . | . | . | . | . | 3 | . | 2 | 4 | 3 | . |
| 24 | *Cancer pagurus* | . | . | . | . | . | . | . | . | . | . | . | . | 2 | . | 2 | . | . | . |
| 25 | *Cereus pedunculatus* | . | . | . | . | . | . | . | . | . | . | . | . | . | . | . | . | . | 2 |
| 26 | *Chaetopterus variopedatus* | . | . | . | . | . | . | . | . | . | . | . | . | . | 1 | 1 | . | 1 | . |
| 27 | *Chamelea gallina* | . | . | . | . | . | . | . | . | . | . | . | . | 1 | . | . | . | . | . |
| 28 | *Chamelea striatula* | . | . | . | . | . | . | . | . | . | . | . | . | . | . | . | . | . | . |
| 29 | *Chlamys varia* | 5,05 | 0,29 | 1,00 | 1,47 | 1,00 | 119,15 | 0,00 | 0,00 | 0,00 | 0,00 | 0,00 | 1 | . | . | . | . | . | . |
| 30 | Ascidia sp. | . | . | . | . | . | . | . | . | . | . | . | . | . | . | 2 | . | . | . |
| 31 | *Conilera cylindracea* | . | . | . | . | . | . | . | . | . | . | . | . | . | 2 | . | . | 1 | . |
| 32 | *Corbula gibba* | 1,02 | 1,45 | 1,50 | 2,23 | 0,11 | 0,68 | 0,00 | 2,58 | 0,00 | 0,00 | 0,00 | 1 | . | . | 1 | . | . | . |
| 33 | *Crangon crangon* | . | . | . | . | . | . | . | . | . | . | . | . | . | . | . | . | . | . |
| 34 | *Crepidula fornicata* | . | . | . | . | . | . | . | . | . | . | . | . | 1 | 1 | . | . | 1 | . |
| 35 | *Phaxas pellucidus* | 2,00 | 1,73 | 1,50 | 5,20 | 0,11 | 0,30 | 0,00 | 0,00 | 1,77 | 0,00 | 0,00 | 2 | 1 | . | . | 1 | . | . |
| 36 | Euclymene sp. | 3,23 | 1,92 | 1,20 | 7,45 | 0,11 | 0,00 | 12,38 | 0,00 | 0,00 | 0,00 | 0,00 | 2 | 2 | 1 | 2 | . | . | . |
| 37 | *Eunice vittata* | . | . | . | . | . | . | . | . | . | . | . | . | . | 2 | . | . | . | . |
| 38 | *Eurynome aspera* | . | . | . | . | . | . | . | . | . | . | . | . | . | . | 1 | . | . | . |
| 39 | Flabelligeridae | 5,10 | 1,74 | 1,20 | 10,63 | 0,11 | 0,00 | 64,82 | 0,36 | 0,00 | 0,36 | 0,00 | 3 | . | 3 | . | . | . | . |
| 40 | Sabellidae | . | . | . | . | . | . | . | . | . | . | . | . | . | . | . | . | . | . |
| 41 | Terebellidae | 4,11 | 1,23 | 1,20 | 6,05 | 0,11 | 0,00 | 0,00 | 0,00 | 0,00 | 0,89 | 0,00 | 2 | . | . | . | . | . | . |
| 42 | *Galathea dispersa* | . | . | . | . | . | . | . | . | . | . | . | . | . | 1 | . | . | . | . |
| 43 | Glycera sp. | . | . | . | . | . | . | . | . | . | . | . | . | . | 2 | 2 | . | 2 | . |
| 44 | *Haploops nirae* | 3,64 | 2,87 | 1,00 | 10,44 | 0,11 | 34,10 | 0,00 | 2,40 | 0,00 | 1430,68 | 0,00 | 3 | . | 1 | 1 | . | 4 | . |
| 45 | *Henricia oculata* | . | . | . | . | . | . | . | . | . | . | . | . | . | 3 | . | . | . | . |
| 46 | *Inachus dorsettentis* | 3,76 | 2,57 | 1,00 | 9,66 | 1,00 | 2,04 | 10272,67 | 352,85 | 28,77 | 34,22 | 1,09 | 3 | . | 2 | 2 | 3 | 3 | . |
| 47 | *Leptopentacta elongata* | . | . | . | . | . | . | . | . | . | . | . | . | 3 | . | . | . | . | 3 |
| 48 | *Limea lascombii* | . | . | . | . | . | . | . | . | . | . | . | . | . | . | . | . | . | . |
| 49 | *Limopsis minuta* | . | . | . | . | . | . | . | . | . | . | . | . | . | . | . | . | . | . |
| 50 | *Liocarcinus navigator* | 3,40 | 1,13 | 1,00 | 3,84 | 1,00 | 0,00 | 0,00 | 0,00 | 3,97 | 0,48 | 96,59 | 1 | . | . | . | . | 3 | . |
| 51 | *Liocarcinus vernalis* | . | . | . | . | . | . | . | . | . | . | . | . | . | . | . | 3 | . | . |
| 52 | *Liocarcinus depurator* | 3,40 | 0,49 | 1,00 | 1,66 | 1,00 | 0,00 | 150,89 | 0,00 | 0,00 | 3,35 | 0,00 | 1 | . | 2 | . | . | . | . |
| 53 | *Liocarcinus holsatus* | 3,40 | 0,79 | 1,00 | 2,68 | 1,00 | 0,00 | 240,56 | 0,00 | 4,13 | 0,00 | 0,00 | 1 | . | . | . | 3 | . | 2 |
| 54 | *Liocarcinus pusillus* | 3,40 | 2,29 | 1,00 | 7,80 | 1,00 | 1,36 | 665,20 | 10,47 | 0,00 | 0,00 | 0,00 | 2 | 3 | 1 | 1 | . | . | . |
| 55 | *Luidia ciliaris* | . | . | . | . | . | . | . | . | . | . | . | . | 3 | . | . | . | . | . |
| 56 | *Lumbrineris tetraura* | 5,84 | 1,89 | 1,20 | 13,24 | 0,11 | 0,00 | 66,74 | 0,84 | 0,00 | 0,00 | 0,00 | 3 | . | 2 | 2 | . | . | . |
| 57 | *Lutraria magna* | . | . | . | . | . | . | . | . | . | . | . | . | . | . | . | . | 1 | . |
| 58 | *Lyonsia norwegica* | . | . | . | . | . | . | . | . | . | . | . | . | 1 | 1 | 1 | . | . | . |
| 59 | *Macropodia rostrata* | . | . | . | . | . | . | . | . | . | . | . | . | . | . | 1 | 3 | . | . |
| 60 | *Maja brachydactyla* | . | . | . | . | . | . | . | . | . | . | . | . | . | . | . | . | . | . |
| 61 | *Maldane glebifex* | 3,23 | 1,63 | 1,20 | 6,30 | 0,11 | 22,04 | 0,00 | 22,04 | 0,00 | 0,85 | 0,00 | 2 | 2 | . | 2 | . | . | . |
| 62 | *Marthasterias glacialis* | . | . | . | . | . | . | . | . | . | . | . | . | 3 | . | 3 | 3 | . | . |
| 63 | Porifera | . | . | . | . | . | . | . | . | . | . | . | . | . | . | . | . | . | . |
| 64 | *Munida rugosa* | . | . | . | . | . | . | . | . | . | . | . | . | . | . | . | . | . | . |
| 65 | *Mysia undata* | . | . | . | . | . | . | . | . | . | . | . | . | . | . | . | . | . | . |
| 66 | *Nassarius reticulatus* | 1,194 | 0,76 | 1,00 | 0,91 | 1,00 | 18,34 | 0,47 | 15,51 | 32,68 | 59,12 | 3,65 | 1 | 2 | 2 | 2 | 2 | 3 | 2 |
| 67 | *Natatolana neglecta* | . | . | . | . | . | . | . | . | . | . | . | . | . | 2 | . | . | . | . |
| 68 | *Necora puber* | . | . | . | . | . | . | . | . | . | . | . | . | 2 | . | . | . | 3 | . |
| 69 | Nemertina sp. | 5,19 | 2,60 | 1,00 | 13,48 | 0,11 | 0,00 | 0,01 | 0,00 | 0,00 | 0,00 | 0,00 | 3 | . | . | . | . | 1 | . |
| 70 | *Nereis pelagica* | . | . | . | . | . | . | . | . | . | . | . | . | . | 2 | . | . | 3 | . |
| 71 | *Notomastus latericeus* | 2,97 | 1,06 | 1,20 | 3,76 | 0,11 | 0,00 | 0,00 | 0,00 | 0,00 | 0,00 | 0,00 | 1 | . | . | . | . | . | . |
| 72 | *Nucula sulcata* | 1,152 | 1,63 | 1,50 | 2,81 | 0,11 | 0,00 | 14,41 | 0,00 | 0,20 | 0,29 | 46,39 | 1 | . | 1 | . | . | . | 1 |
| 73 | Discodoris sp. | . | . | . | . | . | . | . | . | . | . | . | . | . | . | 3 | . | . | . |
| 74 | *Ophiocomina nigra* | 1,95 | 0,57 | 1,00 | 1,11 | 1,00 | 0,00 | 0,00 | 688,36 | 0,00 | 0,00 | 0,00 | 1 | . | . | 2 | . | . | . |
| 75 | *Ophiocotrix fragilis* | . | . | . | . | . | . | . | . | . | . | . | . | . | . | . | . | . | . |
| 76 | *Ophiura albida* | . | . | . | . | . | . | . | . | . | . | . | . | . | . | . | . | . | . |
| 77 | *Ophiura ophiura* | 2,04 | 1,19 | 1,00 | 2,42 | 1,00 | 4,90 | 0,00 | 0,00 | 110,60 | 25,58 | 308,96 | 1 | 3 | . | . | 3 | . | 3 |
| 78 | Orbiniidae | 2,06 | 1,36 | 1,20 | 3,37 | 0,11 | 0,00 | 19,18 | 0,00 | 0,00 | 0,00 | 0,00 | 1 | . | 2 | . | . | . | . |
| 79 | *Owenia fusiformis* | 5,18 | 1,63 | 1,20 | 10,15 | 0,11 | 0,00 | 0,00 | 2,03 | 0,00 | 0,00 | 0,00 | 3 | . | . | 1 | . | . | . |
| 80 | *Pagurus bernhardus* | . | . | . | . | . | . | . | . | . | . | . | . | . | . | . | . | . | . |
| 81 | *Pagurus prideaux* | . | . | . | . | . | . | . | . | . | . | . | . | 2 | 2 | . | . | 3 | . |
| 82 | *Pandalina brevirostris* | . | . | . | . | . | . | . | . | . | . | . | . | . | 2 | 2 | . | 2 | . |
| 83 | *Pectinaria koreni* | . | . | . | . | . | . | . | . | . | . | . | . | . | . | 3 | . | . | . |
| 84 | *Pecten maximus* | . | . | . | . | . | . | . | . | . | . | . | . | 1 | 3 | 1 | . | . | . |
| 85 | Pherusa sp*.* | . | . | . | . | . | . | . | . | . | . | . | . | . | 1 | 1 | . | . | . |
| 86 | *Philocheras sculptus* | . | . | . | . | . | . | . | . | . | . | . | . | . | . | . | . | . | . |
| 87 | *Philocheras trispinosus* | . | . | . | . | . | . | . | . | . | . | . | . | . | 2 | . | 2 | . | 2 |
| 88 | *Philine aperta* | 1,30 | 1,66 | 1,00 | 2,16 | 1,00 | 45,95 | 4,80 | 5,26 | 17,54 | 2,70 | 20,66 | 1 | 3 | 3 | 3 | 4 | . | 1 |
| 89 | *Pisidia longicornis* | 6,69 | 3,38 | 1,00 | 22,62 | 1,00 | 0,00 | 0,00 | 0,00 | 0,00 | 3,13 | 0,00 | 3 | . | . | . | . | 4 | . |
| 90 | *Pododesmus squama* | . | . | . | . | . | . | . | . | . | . | . | . | . | 1 | . | . | . | . |
| 91 | *Pontophilus spinosus* | . | . | . | . | . | . | . | . | . | . | . | . | . | 2 | . | . | . | . |
| 92 | *Pontobdella muricata* | . | . | . | . | . | . | . | . | . | . | . | . | . | 2 | . | . | . | . |
| 93 | *Psammechinus miliaris* | . | . | . | . | . | . | . | . | . | . | . | . | . | . | . | . | . | . |
| 94 | Sabellidae | . | . | . | . | . | . | . | . | . | . | . | . | . | 1 | . | . | . | 2 |
| 95 | Sagartia sp. | . | . | . | . | . | . | . | . | . | . | . | . | . | . | . | . | . | 2 |
| 96 | *Sepia officinalis* | . | . | . | . | . | . | . | . | . | . | . | . | 2 | . | . | 2 | . | . |
| 97 | *Sepiola atlantica* | . | . | . | . | . | . | . | . | . | . | . | . | . | . | . | . | . | . |
| 98 | Sipunculus sp. | . | . | . | . | . | . | . | . | . | . | . | . | . | . | . | . | 1 | . |
| 99 | *Sipunculus nudus* | . | . | . | . | . | . | . | . | . | . | . | . | . | . | 2 | . | . | . |
| 100 | *Spisula elliptica* | . | . | . | . | . | . | . | . | . | . | . | . | . | . | . | . | . | . |
| 101 | *Spisula subtruncata* | 1,89 | 1,63 | 1,50 | 4,63 | 0,11 | 6,15 | 1,00 | 5,69 | 2,36 | 0,00 | 0,00 | 2 | 1 | 3 | 1 | 1 | . | . |
| 102 | *Sternapsis scutata* | 3,13 | 1,55 | 1,20 | 5,85 | 0,11 | 0,00 | 52,89 | 5,62 | 2,95 | 0,04 | 45,25 | 2 | . | 1 | 1 | 1 | . | 1 |
| 103 | *Sthenelais boa* | . | . | . | . | . | . | . | . | . | . | . | . | 2 | 2 | . | . | . | . |
| 104 | *Politapes rhomboides* | 0,85 | 0,50 | 1,50 | 0,63 | 0,11 | 0,00 | 3271,71 | 0,00 | 0,00 | 52,71 | 0,00 | 1 | . | 1 | 1 | . | . | . |
| 105 | *Tellina fabula* | . | . | . | . | . | . | . | . | . | . | . | . | . | . | . | . | . | . |
| 106 | *Terebellides stroemi* | 4,20 | 1,75 | 1,20 | 8,84 | 0,11 | 0,00 | 31,78 | 0,00 | 0,24 | 0,00 | 0,00 | 3 | . | 1 | . | . | . | . |
| 107 | *Thracia pubescens* | . | . | . | . | . | . | . | . | . | . | . | . | . | . | . | . | . | . |
| 108 | *Thyone fusus* | . | . | . | . | . | . | . | . | . | . | . | . | 1 | . | . | . | . | . |
| 109 | *Timoclea ovata* | . | . | . | . | . | . | . | . | . | . | . | . | . | 1 | . | . | . | . |
| 110 | *Turritella communis* | . | . | . | . | . | . | . | . | . | . | . | . | . | . | . | . | . | . |
| 111 | Paguridae sp | 2,83 | 2,49 | 1,00 | 7,05 | 1,00 | 0,00 | 1921,41 | 13,24 | 19,31 | 21,03 | 8,64 | 2 | 1 | 1 | 1 | 5 | 1 | 1 |
| 112 | *Virgularia mirabilis* | . | . | . | . | . | . | . | . | . | . | . | . | . | . | . | 5 | . | 1 |
| 113 | *Xantho pilipes* | . | . | . | . | . | . | . | . | . | . | . | . | . | 2 | . | . | . | . |

**Table S2**: Full description of fish species considered in this study, including: species codes (used for example in two-dimentional δ-spaces), scientific names, size limit in cm (when a filter was needed), and mobility group. Codes definition was: 1 for species moving up to 100 m; 2 for species moving between 100 m and 1 km; 3 for species moving between 1 and 10 km; or 4 for species moving over 10 km in about 3 weeks.

| **Species ID** | **Species scientific names** | **Size limits** | **Mobility groups** |
| --- | --- | --- | --- |
| F1 | *Aphia minuta* | / | 2 |
| F2 | *Arnoglossus laterna* | / | 3 |
| F3 | *Atherina presbyter* | / | 4 |
| F4 | *Buglossidium luteum* | 11 | 3 |
| F5 | *Callionymus lyra* | 8 | 2 |
| F6 | *Ciliata mustela* | / | 1 |
| F7 | *Ctenolabrus rupestris* | / | 1 |
| F8 | *Dicologlossa cuneata* | 7 | 3 |
| F9 | *Diplecogaster bimaculata bimaculata* | 4 | 1 |
| F10 | *Engraulis encrasicolus* | / | 4 |
| F11 | *Gobius niger* | 10 | 1 |
| F12 | *Gobius spp.* | NA | NA |
| F13 | *Gaidropsarus vulgaris* | / | 3 |
| F14 | *Labrus bergylta* | / | 3 |
| F15 | *Lepidorhombus whiffiagonis* | 8 | 3 |
| F16 | *Lophius piscatorius* | / | 3 |
| F17 | *Merlangius merlangus* | 16 | 4 |
| F18 | *Merluccius merluccius* | excluded | 4 |
| F19 | *Pleuronectes platessa* | / | 3 |
| F20 | *Pomatoschistus minutus* | 5 | 2 |
| F21 | *Scyliorhinus canicula* | excluded | 4 |
| F22 | *Solea senegalensis* | 20 | 3 |
| F23 | *Solea solea* | 17 | 3 |
| F24 | *Sprattus sprattus* | 10 | 4 |
| F25 | *Syngnathus acus* | / | 1 |
| F26 | *Syngnathus rostellatus* | / | 1 |
| F27 | *Trachurus trachurus* | 14 | 4 |
| F28 | *Trisopterus luscus* | 17 | 4 |
| F29 | *Trisopterus minutus* | 12 | 4 |
| F30 | *Zeus faber* | 10 | 3 |

**
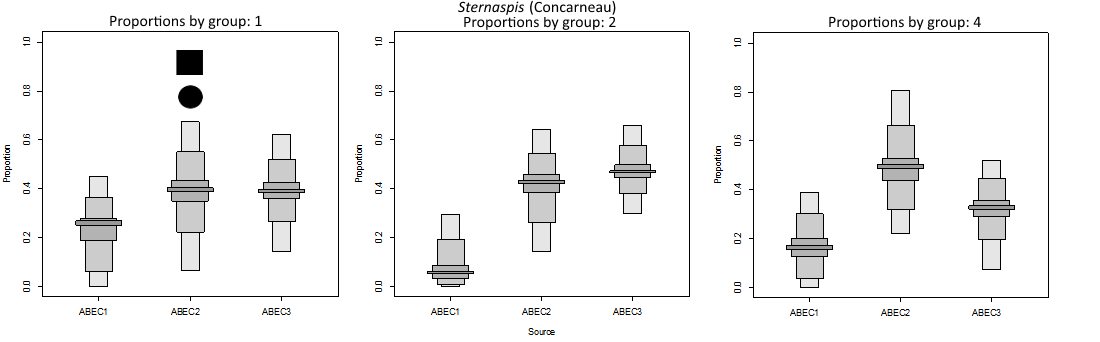
**

**
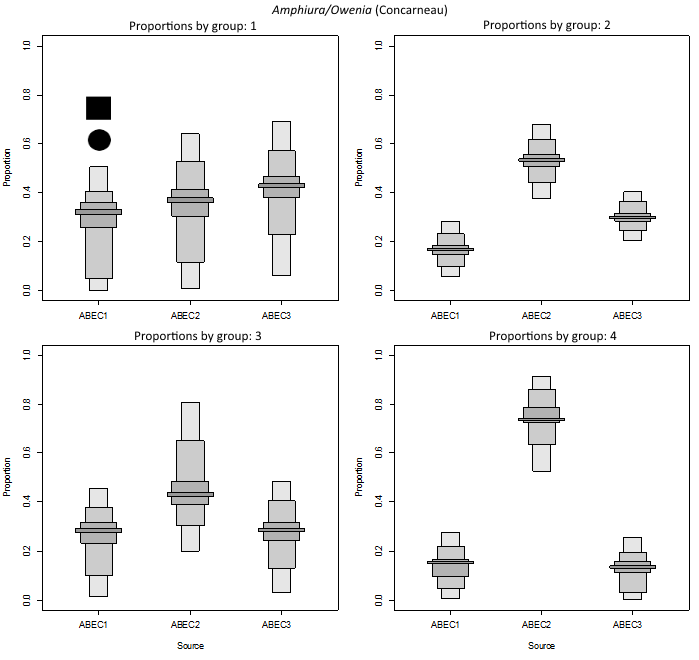
**

**
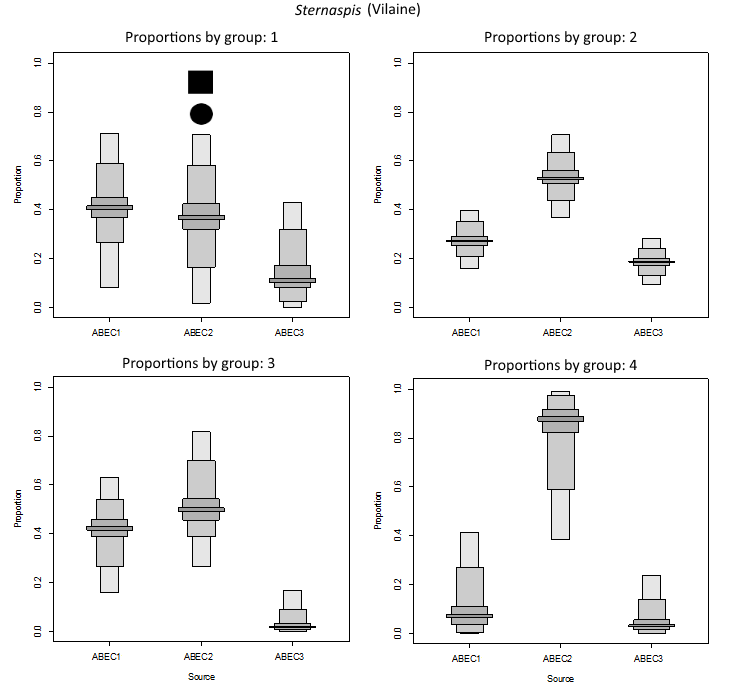

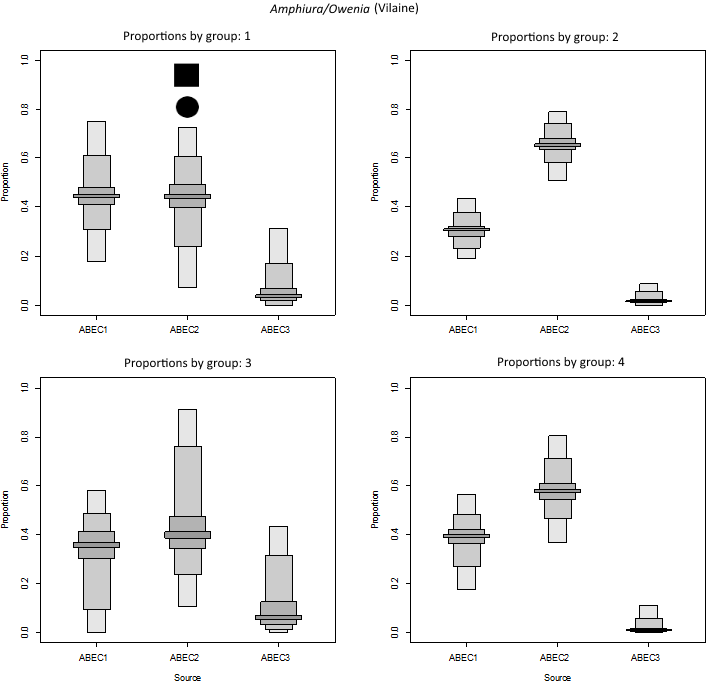
**

**Figure S1:** Contribution of the ABEC groups to the diets of predators in *Sternaspis* and *Amphiura/Owenia* habitats of the Bay of Concarneau and the Bay of Vilaine, using SIAR mixing model. Box plots illustrate the 25th, 50th and 75th percentiles; the whiskers indicate the 10th and 90th percentiles. The details of ABEC-based sources’ compositions (i.e. ABEC groups 1 to 3) are given in the Appendix Table S1. From top left to bottom are displayed the results for: Mobility group 1 (up to 100 m), mobility group 2 (between 100 m and 1 km), and mobility group 4 (above 10 km). The black circle indicates the ABEC source associated to the highest accessible biomass and the black square indicates the source with the highest energetic supply (E_i_ × π_i_ × R_i_).


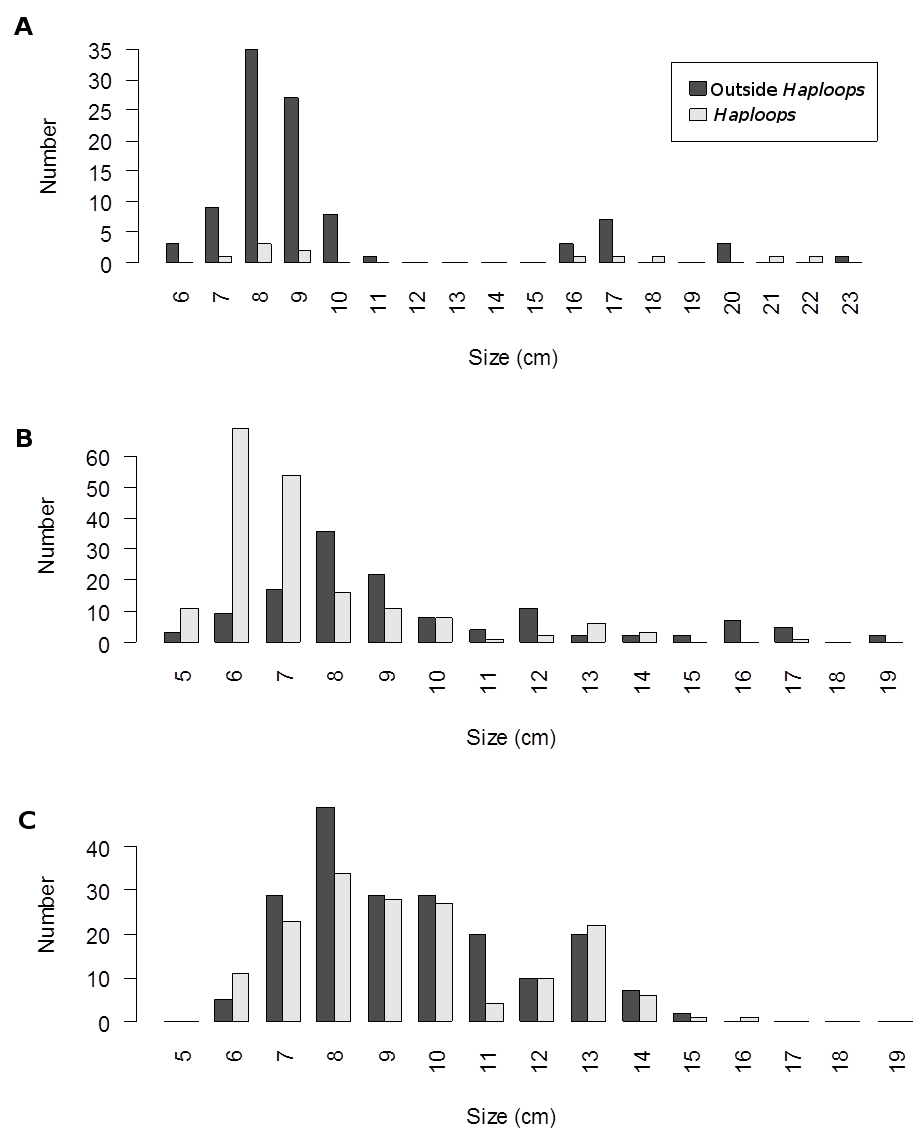


**Figure S2:** Size spectra per mobility groups: **A.** Mobility group 3 (between 1 and 10 km) for the Bay of Concarneau only. **B.** Mobility group 4 (above 10 km) for the Bay of Concarneau. **C.** Mobility group 4 for the Bay of Vilaine. Each size spectrum considered the individuals of *Haploops* habitat (in white), and outside *Haploops* habitats together (in black).
